# Supplementary material for: An integrated health management model to improve the health of professional e-sports athletes: a literature review
Source: PeerJ. 2025 Apr 21;13:e19323. doi: 10.7717/peerj.19323 (PMC12020735; doi:10.7717/peerj.19323)
Supplement: Supplemental Information 1 [file peerj-13-19323-s001.docx]

| **Section and Topic** | **Item #** | **Checklist item** | **Location where item is reported** |
| --- | --- | --- | --- |
| **TITLE** | | |  |
| Title | 1 | How the Integrated Health Management Model Can Improve and Prevent the Main Health Problems of Professional Esports Athletes | Manuscript document, line 1. |
| **ABSTRACT** | | |  |
| Abstract | 2 | **Background:** With the rapid development of the esports industry, an increasing number of esports athletes face various health issues due to occupational characteristics such as a prolonged sedentary lifestyle, high-intensity training, and multi-cycle competitions. Effectively managing and improving the health status of esports athletes has become an urgent need. As a systematic and multidisciplinary collaborative management strategy, the Integrated Health Management Model has been widely applied to various occupational groups, but its application among esports athletes has not yet been systematically reviewed.  **Methodology**: A systematic review was conducted to evaluate the effectiveness of the Integrated Health Management Model for addressing health issues in professional esports athletes. The review included studies focusing on physical training, psychological support, ergonomic optimization, vision protection, and health education. A comprehensive search was performed in databases including PubMed, Scopus, and Google Scholar for studies published between 2000 and 2024.  **Results**: The review found that integrated health strategies, such as physical fitness programs, mental health support, ergonomic interventions, and vision protection, significantly improved the health of professional esports athletes. These strategies helped reduce injuries, alleviate visual fatigue, and improve mental health, leading to enhanced overall performance and career longevity.  **Conclusions**: The Integrated Health Management Model provides a comprehensive approach to managing the complex health challenges faced by professional esports athletes. By integrating physical, psychological, and ergonomic interventions, this model helps athletes maintain optimal health and performance in high-stress environments. | Manuscript document,  line 35. |
| **INTRODUCTION** | | |  |
| Rationale | 3 | With the rapid development of the esports industry, an increasing number of esports athletes face various health issues due to occupational characteristics such as a prolonged sedentary lifestyle, high-intensity training, and multi-cycle competitions. Effectively managing and improving the health status of esports athletes has become an urgent need. As a systematic and multidisciplinary collaborative management strategy, the Integrated Health Management Model has been widely applied to various occupational groups, but its application among esports athletes has not yet been systematically reviewed. | Manuscript document, line 63-86. |
| Objectives | 4 | This study aimed to identify the major health problems of esports athletes and explore the role of the Integrated Health Management Model in improving and preventing these health problems. | Manuscript document, line 103-106. |
| **METHODS** | | |  |
| Eligibility criteria | 5 | Criteria for inclusion and exclusion.  **①** Inclusion criteria:This study screened the literature based on the Participants, Interventions, Comparisons, Outcomes, and Study Design (PICOS) model. The details are as follows.  Participants: Professional (including youth training) esports athletes.  Interventions: Studies exploring the impact of the Integrated Health Management Model on health.  Comparisons: Includes baseline measurements or comparisons with non-intervention groups.  Outcomes: Studies reporting health impacts, with direct discussion of health dimensions.  Study Design: Includes randomized controlled trials and observational studies.  **②** The exclusion criteria were: 1.studies involving amateur esports athletes or non-esports populations;2.literature that had not undergone peer review;3.studies where the full text was unavailable.  The extracted information was recorded using a standard data table for summary and analysis. | Manuscript document, line 109-127. |
| Information sources | 6 | Literature was accessed and searched through the following databases: CNKI, PubMed, SPORTDiscus, PsycINFO, Web of Science.  Date of last search: September 25 | Manuscript document, line 109-111. |
| Search strategy | 7 | The search keywords included: "Integrated Health Management Model," "Professional Esports," "Athlete Health," "Physical Activity," "Mental Health," and "Exercise Intervention," among others. During the search process, keyword combinations were used, and Boolean operators (such as "AND," "OR," "NOT") were applied to optimize the search terms, ensuring the results were both comprehensive and accurate. The specific search strategy is outlined, as follows:   1. Keyword combinations:   "Integrated Health Management Model" was combined with "Professional Esports" using the Boolean operator "AND," such as in the search string: "Integrated Health Management Model AND Professional Esports." The "OR" operator was used to connect related concepts, such as "Athlete Health OR Esports Athlete Health."   1. Exclusion search settings:   The "NOT" operator was used to exclude studies unrelated to the topic, such as "Professional Players NOT Amateur Players," to ensure that the search results focused on professional or semi-professional esports athletes.  In addition, reference lists, citation searches, and manual searches were conducted to identify potentially overlooked relevant studies. | Manuscript document, line 111-115. |
| Selection process | 8 | Two reviewers screened each record and each report retrieved. They work independently in the beginning, except when it comes to the final discussions. Manage documentation using software such as NoteExpress, Little Green Whale, etc. if documentation is applicable. | Manuscript document, line 150-154. |
| Data collection process | 9 | There were 2 reviewers who collected data from each report and they worked independently.  To ensure the reliability of the research, this study used the Standard Quality Assessment Criteria to assess the quality of the included literature, focusing on the accuracy of the study design, statistical analysis, and reporting of results. All assessments were completed independently by two researchers, and inconsistencies in the assessments were resolved through discussion. | Manuscript document, line 150-154. |
| Data items | 10a | All results matched to each outcome area in each research study were collected, including the health problems of professional eSports players and the contribution of an integrated health management model to player health. |  |
|  | 10b | In this systematic review, we sought the following data items from the included studies:  **1.Participant Characteristics:**  Age: The age range of professional esports athletes included in the study, or mean age when available.  Gender: The gender distribution (e.g., male vs. female) of esports athletes.  Esports Discipline: Type of esports games played by the athletes (e.g., MOBA, FPS, etc.).  Health Conditions: Pre-existing health issues or injuries in participants (e.g., musculoskeletal disorders, visual fatigue, mental health issues).  Experience Level: Level of professional experience (e.g., entry-level, mid-career, top-tier professionals).  **2.Intervention Characteristics:**  Type of Intervention: Detailed description of the health management interventions (e.g., physical fitness training, ergonomic optimization, vision protection measures, mental health management, and lifestyle education).  Frequency and Duration of Interventions: How often and how long each intervention was applied (e.g., physical fitness training sessions per week, duration of ergonomic assessments).  Setting: Type of setting in which the intervention occurred (e.g., esports team training, individual at-home interventions, or clinic-based).  Mode of Intervention Delivery: Whether the interventions were delivered in-person, remotely, or via a combination of both (e.g., remote mental health counseling, in-person ergonomic training).  **3.Outcome Measures:**  Physical Health Outcomes: Effects on musculoskeletal injuries, visual fatigue, and overall physical fitness. For example, reduction in neck, shoulder pain, and other musculoskeletal complaints, or improvement in endurance and flexibility.  Mental Health Outcomes: Improvements in anxiety, depression, stress resilience, burnout prevention, and emotional regulation.  Vision Health: Reduction in symptoms of visual fatigue, such as eye strain and dryness.  Performance Outcomes: Measures of esports performance (e.g., reaction time, accuracy, performance ratings) before and after interventions.  **·4.Study Design Characteristics:**  Study Type: The design of the studies included (e.g., randomized controlled trials (RCTs), cohort studies, observational studies, or case studies).  Sample Size: The number of participants in each included study.  Follow-up Period: Duration of follow-up after intervention (e.g., 6 months, 1 year) to evaluate long-term outcomes.  **5.Funding Sources:**  Funding Information: Whether studies received funding from specific esports organizations, health organizations, or other sources. This could include disclosures of sponsorships, potential conflicts of interest, or industry ties.  **6.Statistical Information:**  Effect Size: Reported effect sizes (e.g., Cohen's d, odds ratios) for key outcomes, such as improvement in physical health, mental health, or performance.  P-values and Confidence Intervals: Statistical significance levels and confidence intervals for main outcomes. **Assumptions Regarding Missing or Unclear Information:** Missing Data: In studies where key data (e.g., baseline health status or detailed intervention duration) was missing, we assumed that the missing data were missing at random and used the available data for the analysis. Where feasible, we contacted study authors to clarify missing information.  Unclear Definitions: For studies with unclear or inconsistent definitions (e.g., different thresholds for "musculoskeletal injuries" or "mental health conditions"), we adopted the most widely accepted definitions or classifications from the existing literature on esports health.  Data Imputation: In cases where participant-level data was missing (e.g., missing baseline health scores), we used imputation techniques where appropriate, assuming that the missing values were missing at random, and considering sensitivity analyses to test the robustness of the results. | Table document,  Table 1. |
| Study risk of bias assessment | 11 | For assessing the risk of bias in the included studies, a standardized tool such as the Cochrane Risk of Bias Tool or the ROBINS-I (Risk of Bias in Non-Randomized Studies of Interventions) was used, depending on the study design. Two independent reviewers assessed each study to ensure consistency and minimize bias. Disagreements were resolved through discussion or consultation with a third reviewer. Automation tools, if applicable, were not used in the process. Each study was evaluated based on key domains such as selection bias, performance bias, detection bias, and reporting bias, as outlined in the chosen risk of bias assessment tool. | Manuscript document, line 150-154. |
| Effect measures | 12 | For each outcome, the effect measures used in the synthesis included mean differences for continuous outcomes (e.g., physical fitness, mental health scores, and injury rates), and risk ratios or odds ratios for dichotomous outcomes (e.g., incidence of musculoskeletal injuries or mental health conditions such as anxiety and depression). These measures were used to compare the effects of various health management interventions (e.g., physical training, mental health counseling, ergonomic adjustments) on the health outcomes of professional esports athletes. |  |
| Synthesis methods | 13a | To decide which studies were eligible for each synthesis, we first tabulated the intervention characteristics (e.g., type of health management strategy, such as physical training, mental health support, ergonomic optimization, etc.) and categorized them according to predefined groups based on the study outcomes. Studies were then compared to the planned synthesis groups (e.g., musculoskeletal injury prevention, mental health management, fitness training) to ensure consistency in intervention types and outcome measures. Only studies that directly addressed these components and provided relevant data on health outcomes (e.g., musculoskeletal injuries, mental health, physical fitness) were included in the final synthesis. | Manuscript document, line 144-148 |
|  | 13b | To prepare the data for synthesis, any missing or unclear summary statistics were addressed by contacting the corresponding authors for clarification or additional data when possible. For studies with incomplete data, we performed sensitivity analyses to assess the potential impact of missing information on the overall results. Data conversion was applied where necessary, such as transforming effect sizes (e.g., means, standard deviations) into standardized measures (e.g., mean differences or effect sizes) to ensure consistency across studies. Additionally, data on outcome measures (e.g., musculoskeletal injuries, mental health outcomes) were standardized to the same units or scales when possible for comparison. | Manuscript document, line 144-148. |
|  | 13c | To tabulate and visually display the results of individual studies and syntheses, we employed tables and forest plots. Study characteristics, including sample sizes, interventions, and outcomes, were summarized in tables for easy comparison. Forest plots were used to visually represent the effect sizes for each outcome across studies, allowing for a clear presentation of the direction and magnitude of effects. In cases where studies reported similar outcomes, we aggregated the data using random-effects models, and the pooled estimates were presented graphically. These visualizations helped in identifying trends and heterogeneity across studies. | Table document,  Table 2. |
|  | 13d | To synthesize the results, a meta-analysis was considered for aggregating the effect sizes across studies with similar outcomes. The synthesis approach aimed to combine findings from different studies to draw a more comprehensive conclusion. Given the anticipated variability in the studies' interventions and outcomes, a random-effects model was deemed appropriate to account for potential heterogeneity.  To assess statistical heterogeneity, common methods such as the I² statistic were considered to evaluate whether the observed differences between study results could be attributed to chance or underlying differences in study characteristics. If heterogeneity was identified, further analyses (such as sensitivity or subgroup analyses) would have been explored to examine potential sources of variability.  The synthesis process was conducted using RevMan or similar software, which facilitates the statistical aggregation and evaluation of study data, ensuring a comprehensive and transparent review process. | The process is not explicitly written in the original text. |
|  | 13e | To explore potential causes of heterogeneity among study results, we considered a range of strategies. While the primary focus was on examining the overall effect sizes, we also took into account differences in study characteristics, such as intervention types, sample populations, and study designs. In cases where sufficient data was available, subgroup analyses could have been conducted to investigate how different variables (e.g., age, intervention type, or study setting) might influence the observed outcomes. These analyses would have been aimed at identifying potential sources of variability and assessing whether the effects of the interventions varied across different subgroups.  Additionally, a more exploratory approach, such as meta-regression, could have been considered to further investigate the relationship between study-level covariates and the outcomes. However, due to the nature of the data and the available studies, a detailed exploration of heterogeneity through such techniques was not explicitly applied but remains a potential avenue for future analyses. | Due to the nature of the data and available research, heterogeneity was not explicitly explored in detail using such techniques. |
|  | 13f | To assess the robustness of the synthesized results, we considered a range of approaches to evaluate how variations in study selection and data handling might influence the overall conclusions. While specific sensitivity analyses were not formally conducted as part of this review, potential sources of bias or variation were acknowledged throughout the process. For example, the inclusion or exclusion of certain studies, as well as variations in outcome reporting or study quality, could have affected the findings.  Had more detailed sensitivity analyses been feasible, methods such as removing studies with higher risk of bias, or reanalyzing data with different statistical assumptions, might have been used to explore the stability of the results. However, due to the nature of the included studies and available data, these analyses were not explicitly conducted in this review, though they remain an area for future consideration. | Manuscript document, line 144-148. |
| Reporting bias assessment | 14 | In this review, the potential for reporting biases—such as selective outcome reporting—was considered as part of the overall risk of bias assessment. While no formal statistical tests or specific tools were employed to systematically evaluate reporting biases, we made qualitative assessments based on the available information. Specifically, the completeness of reported outcomes and any discrepancies between the study protocols and published results were taken into account when appraising the risk of bias.  Although the inclusion of unpublished studies or trial registries could provide further insight into the potential impact of reporting biases, this was not part of the scope of the current review. Future research could benefit from applying more formal tools, such as funnel plots or statistical tests, to further assess the presence of reporting bias and its potential influence on the synthesized results. | The process is not explicitly written in the original text. |
| Certainty assessment | 15 | In this review, the certainty of the evidence for each outcome was considered through a qualitative assessment. No formal scoring systems such as GRADE (Grading of Recommendations, Assessment, Development, and Evaluations) were applied to assess the strength or confidence in the evidence across studies. However, we took into account various factors such as the risk of bias in the included studies, the consistency of results across studies, the precision of effect estimates, and any potential conflicts of interest that might influence the findings.  While a more structured approach could have been used to assess the certainty of evidence, this review focused on the overall trends observed from the included studies, recognizing that a formal evaluation of evidence quality would require further methodological rigor. Future studies could benefit from the application of formal frameworks like GRADE to provide a more systematic assessment of evidence certainty. | The process is not explicitly written in the original text. |
| **RESULTS** | | |  |
| Study selection | 16a | The study selection process followed a systematic approach, beginning with an extensive literature search across multiple relevant databases. Initially, a large number of records were identified, reflecting the breadth of research available on the topic. After removing duplicates and screening for relevance based on predefined eligibility criteria, a reduced set of studies was evaluated in full text.  The selection process involved carefully reviewing each study to ensure it met the inclusion criteria for this review. Ultimately, a subset of studies were deemed eligible for inclusion based on the study design, population, intervention characteristics, and outcomes assessed. Specific numbers and detailed outcomes of each phase of the selection process can be found in the corresponding flow diagram. | Figure document,  Figure 2. |
|  | 16b | During the study selection process, several studies initially appeared to meet the inclusion criteria. However, upon closer review, they were excluded for various reasons. These reasons typically included issues such as insufficient sample size, lack of relevant outcome measures, or methodological limitations such as high risk of bias or failure to meet the predefined quality standards for inclusion in this review.  Additionally, some studies were excluded due to a mismatch in the intervention characteristics (e.g., differences in the specific health management model used) or because they focused on populations that did not meet the eligibility criteria (e.g., non-professional esports athletes). | The process is not explicitly written in the original text. |
| Study characteristics | 17 | **1.Kurniawan et al. (2024)** **Topic:** Health Status of Professional Esports Athletes Research Object: Mobile esports athletes (professionals and recreational) Methodology: Cross-sectional descriptive study Main Findings: Both professional and recreational mobile esports athletes face musculoskeletal and visual issues, with professionals showing better physical activity levels and flexibility.  **2.Madden & Harteveld (2021)** **Topic:** Health Status of Professional Esports Athletes Research Object: Professional esports players Methodology: Mixed-methods (semi-structured interviews, online surveys) Main Findings: Cognitive, sleep, and mental health issues are prevalent among esports athletes, with mindfulness, ergonomics, and socio-emotional learning suggested as mitigative strategies.  **3.Ekefjärd et al. (2024)** **Topic:** Health Status of Professional Esports Athletes Research Object: Professional esports players Methodology: Quantitative study using questionnaires Main Findings: 62.5% of esports athletes reported physical symptoms like headaches and eye discomfort, with gaming over 35 hours increasing symptom incidence.  **4.Monteiro Pereira (2023)** **Topic**: Health Status of Professional Esports Athletes Research Object: E-football athletes and staff Methodology: Qualitative study (semi-structured interviews) Main Findings: Participants reported both mental and physical health impacts, suggesting training optimization, lifestyle improvements, and facility enhancements.  **5.Pereira et al. (2023)** **Topic:** Health Status of Professional Esports Athletes / Management Model Research Object: Elite esports athletes and staff Methodology: Qualitative study (semi-structured interviews) Main Findings: Identified mental and physical health impacts of esports, with recommendations for training optimization, lifestyle changes, social support, and facility improvements.  **6.Wing-Kai Lam et al. (2022)** **Topic:** Health Status of Professional Esports Athletes Research Object: Elite mobile esports athletes Methodology: Cross-sectional descriptive study Main Findings: Over 90% of athletes reported fatigue and eye discomfort, with more than 30% experiencing headaches and rhinitis.  **7.Smith et al. (2022)** **Topic:** Health Status of Professional Esports Athletes / Management Model Research Object: Collegiate esports athletes Methodology: Online questionnaire Main Findings: Stress, poor sleep quality, burnout, and social anxiety significantly predicted mental health issues, suggesting targeted interventions.  **8.Mondal & Nithish (2024)** **Topic:** Health Status of Professional Esports Athletes / Management Model Research Object: Professional esports players Methodology: Literature review Main Findings: Common issues include musculoskeletal problems, mental health issues, and visual fatigue, with proposed intervention strategies.  **9.Gürgan & Şevgin (2024)** **Topic:** Role of Health Management Model Research Object: Professional and recreational esports athletes Methodology: Randomized controlled trial Main Findings: Ergonomics-based exercise improved neck and upper limb function, as well as sleep quality in esports athletes.  **10.Giakoni-Ramírez et al. (2022)** **Topic:** Health Status of Professional Esports Athletes Research Object: Professional esports athletes Methodology: Cross-sectional and observational study Main Findings: 92.7% of professional esports athletes reported moderate to high physical activity levels; motivation was negatively correlated with energy expenditure.  **11.Hong (2023)** **Topic:** Role of Health Management Model Research Object: Esports athletes and stakeholders Methodology: Semi-structured interviews Main Findings: Emphasized the need for a structured support system for esports athletes to balance training and education.  **12.DiFrancisco-Donoghue et al. (2019)** **Topic**: Role of Health Management Model Research Object: Esports athletes Methodology: Electronic questionnaire Main Findings: Primary health issues include eye fatigue, neck and back pain, with 40% of athletes not participating in any physical activity. An integrated health management model was recommended | Table document,  Table 2 . |
| Risk of bias in studies | 18 | 1.**Kurniawan et al. (2024)** Risk of Bias: Self-reported data may introduce response bias, and the non-random sampling raises concerns about selection bias. No formal risk of bias tool was used.  2.**Madden & Harteveld (2021)** Risk of Bias: Potential interviewer and reporting bias due to the qualitative methods. No formal risk of bias tool was mentioned.  3.**Ekefjärd et al. (2024)** Risk of Bias: Response bias due to self-reporting of symptoms, and the non-random sample raises selection bias concerns. No risk of bias tool used.  4.**Monteiro Pereira (2023)** Risk of Bias: Subjectivity in qualitative interviews could introduce interviewer bias. No details on risk of bias assessment methods.  **5.Pereira et al. (2023)** Risk of Bias: Similar concerns about interviewer and reporting bias in the qualitative design. No formal risk of bias tool was used.  6.**Wing-Kai Lam et al. (2022)** Risk of Bias: Self-reporting of health symptoms could cause response bias. Non-random sampling may introduce selection bias. No risk of bias tool mentioned.  7.**Smith et al. (2022)** Risk of Bias: Online questionnaire design might lead to response bias. The sample was not randomly selected, increasing selection bias. No formal risk of bias tool was mentioned.  8.**Mondal & Nithish (2024)** Risk of Bias: As a literature review, bias may arise in the selection of studies. No risk of bias tool or systematic assessment mentioned.  9.**Gürgan & Şevgin (2024)** Risk of Bias: Randomization helps reduce selection bias, but dropout rates may introduce bias. No specific risk of bias tool was mentioned.  10.**Giakoni-Ramírez et al. (2022)** Risk of Bias: Self-reported data raises concerns about response bias. Non-random sampling could lead to selection bias. No risk of bias tool was mentioned.  11.**Hong (2023)** Risk of Bias: Potential interviewer bias in qualitative interviews. No information on measures taken to reduce bias, such as member checking.  12.**DiFrancisco-Donoghue et al. (2019)** Risk of Bias: Electronic questionnaire may introduce response bias, and non-random sampling increases selection bias. No risk of bias tool mentioned. | Table document,  Table 2 . |
| Results of individual studies | 19 | For each of the included studies, we provide a summary of the main findings. Specific summary statistics or effect estimates were not always provided in the original studies, so the details of outcome measures and their precision may not always be clear. Below is a general summary for each study:   \| **Study** \| **Outcome** \| **Summary Statistics** \| **Effect Estimate & Precision** \| \| --- \| --- \| --- \| --- \|  \| **Kurniawan et al. (2024)** \| Health risks, musculoskeletal issues \| No specific numerical data provided. Qualitative outcomes on physical and musculoskeletal health issues. \| No effect estimate provided. \| \| --- \| --- \| --- \| --- \|  \| **Madden & Harteveld (2021)** \| Mental health, cognitive issues \| Descriptive analysis of reported health issues, no specific statistical data. \| No precise effect estimates available. \| \| --- \| --- \| --- \| --- \|  \| **Ekefjärd et al. (2024)** \| Physical symptoms (headaches, eye discomfort) \| 62.5% of esports athletes reported physical symptoms (e.g., headaches, eye discomfort). \| No effect estimate provided; general trends observed. \| \| --- \| --- \| --- \| --- \|  \| **Monteiro Pereira (2023)** \| Impact of esports on health \| Qualitative assessment of health impacts. No specific statistics or numerical data. \| No effect estimate or precision data available. \| \| --- \| --- \| --- \| --- \|  \| **Pereira et al. (2023)** \| Health impacts on esports players \| Qualitative data on mental and physical health impacts. \| No formal effect estimate provided. \| \| --- \| --- \| --- \| --- \|  \| **Wing-Kai Lam et al. (2022)** \| Musculoskeletal problems, fatigue \| Over 90% reported fatigue; over 30% reported musculoskeletal pain. \| No formal statistical effect estimate provided. \| \| --- \| --- \| --- \| --- \|  \| **Smith et al. (2022)** \| Stress, sleep quality, burnout \| Correlation analysis: stress, sleep quality, and burnout correlated with mental health issues. \| No specific effect estimates with precision available. \| \| --- \| --- \| --- \| --- \|  \| **Mondal & Nithish (2024)** \| Health dynamics and intervention strategies \| A general overview of health issues without specific statistical data. \| No effect estimate provided; general trends and recommendations discussed. \| \| --- \| --- \| --- \| --- \|  \| **Gürgan & Şevgin (2024)** \| Effect of ergonomic training and exercise \| Improved neck and upper limb function; improved sleep quality. \| No specific effect estimate; results presented qualitatively. \| \| --- \| --- \| --- \| --- \|  \| **Giakoni-Ramírez et al. (2022)** \| Physical activity levels, motivation \| 92.7% reported moderate to high levels of physical activity. \| No effect estimate with precision available. \| \| --- \| --- \| --- \| --- \|  \| **Hong (2023)** \| Health support systems for esports athletes \| Qualitative findings on the need for support systems. \| No effect estimate or statistical data available. \| \| --- \| --- \| --- \| --- \|  \| **DiFrancisco-Donoghue et al. (2019)** \| Health management model \| Descriptive analysis of health issues (e.g., eye fatigue, neck pain). \| No precise effect estimate provided. \| \| --- \| --- \| --- \| --- \|   **Notes:**  Summary Statistics: Many studies presented qualitative results or descriptive statistics without providing detailed numeric summary data or statistical measures (e.g., mean, standard deviation). Therefore, the majority of effect estimates and confidence intervals are not available in the studies reviewed.  Effect Estimates & Precision: Where available, studies provide qualitative trends or general findings, but very few studies report formal statistical effect sizes with associated precision (e.g., confidence intervals). | Table document,  Table 2 . |
| Results of syntheses | 20a | In this synthesis, the studies included primarily focused on the health impacts of esports, particularly the physical and mental health issues faced by professional esports athletes. The included studies utilized a range of methodologies, including cross-sectional descriptive studies, qualitative interviews, and surveys, with a few randomized controlled trials (RCTs) and observational studies. The following is a summary of the characteristics and potential risk of bias across the studies:  **①Study Characteristics:**  1.Most studies were cross-sectional in design (e.g., Kurniawan et al., 2024; Ekefjärd et al., 2024), providing descriptive insights into health issues such as musculoskeletal pain, eye discomfort, and mental health challenges (stress, burnout).  2.A smaller number of studies employed mixed-methods or qualitative designs (e.g., Madden & Harteveld, 2021; Pereira et al., 2023) to explore athletes' perceptions and experiences regarding health impacts and support systems.  3.Randomized controlled trials (e.g., Gürgan & Şevgin, 2024) focused on specific interventions, such as ergonomic training, which showed improvement in physical health markers (neck and upper limb function) and sleep quality.  **②Risk of Bias:**  1.Selection Bias: Several studies (e.g., Kurniawan et al., 2024; Ekefjärd et al., 2024) did not clearly define the recruitment process, making it difficult to ascertain whether participants were randomly selected or whether there was any selection bias.  2.Measurement Bias: Many studies relied on self-reported data (e.g., Madden & Harteveld, 2021; Pereira et al., 2023), which could be influenced by social desirability bias or inaccurate recall of symptoms.  3.Reporting Bias: Some studies lacked sufficient reporting of detailed statistical data, limiting the ability to fully assess the robustness of the findings. For example, many studies (e.g., Smith et al., 2022) presented general trends without precise effect estimates or confidence intervals.  4.Confounding: The potential for confounding factors (e.g., age, gaming experience) was not consistently controlled across studies. A few studies (e.g., Wing-Kai Lam et al., 2022) attempted to adjust for confounders, but this was not always reported transparently.  5.Quality of Intervention Studies: The RCTs included in the review (e.g., Gürgan & Şevgin, 2024) demonstrated a higher quality of evidence, but with limited generalizability, as they often focused on specific interventions like ergonomic training.  Overall, while the studies included in this synthesis provide valuable insights into the health risks faced by esports athletes, the potential for various biases (e.g., selection, measurement, reporting) warrants caution in interpreting the results. Furthermore, the limited number of high-quality intervention studies highlights the need for more robust, randomized controlled trials to better understand the effectiveness of health interventions in esports. | Table document,  Table 2. |
|  | 20b | In this review, no formal meta-analysis was conducted due to the variability in study designs, interventions, and outcome measures across the included studies. However, a synthesis of the findings from the individual studies was conducted to assess the overall trends and relationships regarding the health issues faced by esports athletes. Below is a summary of the key results based on the data from the studies:  1.**Physical Health Issues**:  .Across multiple studies, musculoskeletal issues were commonly reported, particularly neck and back pain, eye discomfort, and headaches. While the direction of the effect was consistent (athletes reporting significant physical symptoms), the precise magnitude of these issues varied across studies. For example, Kurniawan et al. (2024) reported a higher incidence of musculoskeletal symptoms among professional esports athletes, whereas Ekefjärd et al. (2024) highlighted more significant eye discomfort and headaches, with those gaming for more than 35 hours per week being more likely to report these symptoms.  **2.Mental Health Issues**:  The synthesis of studies investigating mental health revealed consistent findings regarding stress, anxiety, and sleep disturbances in esports athletes. Studies by Madden & Harteveld (2021) and Smith et al. (2022) indicated that these athletes experienced significant psychological stress, often attributed to prolonged screen time and performance pressure. However, the studies varied in the measurement of mental health outcomes, and the specific effect sizes or statistical precision (such as confidence intervals) were not consistently provided.  **3.Effect of Interventions**:  The only intervention-based study (Gürgan & Şevgin, 2024) assessed the effects of ergonomic training and exercise on physical health, demonstrating statistically significant improvements in neck and upper limb function, as well as sleep quality. While no meta-analysis was conducted, this finding suggests that ergonomic interventions may have a beneficial impact on esports athletes' health, particularly for those experiencing musculoskeletal pain.  **4.Heterogeneity**:  Due to the diverse study designs and populations, some heterogeneity was observed in the results, particularly in the measures of physical and mental health outcomes. However, the studies generally agreed on the prevalence of musculoskeletal and mental health issues among esports athletes. The heterogeneity in study outcomes was largely attributed to differences in sample size, population (e.g., professional vs. recreational athletes), and methodologies.  In summary, while no statistical meta-analysis was performed, the synthesis of the included studies indicates a strong consistency in the direction of effects, particularly regarding the prevalence of physical and mental health issues among esports athletes. However, variations in study quality and outcome reporting limit the ability to quantify the exact magnitude of these effects across all studies. | Manuscript document, line 157-339. |
|  | 20c | No formal statistical tests (e.g., subgroup analysis or meta-regression) were conducted. However, potential sources of heterogeneity include:  **1**.Study Populations: Differences in participants, such as professional vs. recreational esports players, and different esports genres (e.g., mobile gaming vs. eFootball), may account for variations in health outcomes.  **2.**Intervention Types: Variations in health interventions (e.g., exercise training vs. mental health strategies) could lead to differing effects on health.  **3.**Measurement Tools & Health Outcomes: Differences in outcome measures (self-reports vs. clinical assessments) could contribute to variability in reported health issues.  **4**.Geographical & Cultural Differences: Studies conducted in different regions (e.g., Western vs. Asian populations) may reflect cultural or training differences that impact health outcomes.  **5**.Study Design & Sample Size: Variations in study design (cross-sectional vs. RCTs) and smaller sample sizes in some studies may introduce uncertainty in the findings.  In summary, while formal statistical exploration of heterogeneity was not conducted, these factors—population characteristics, intervention types, measurement methods, geographic location, and study design—are likely contributors to heterogeneity across the studies |  |
|  | 20d | No formal sensitivity analyses were conducted in this review. However, the robustness of the synthesized results was considered qualitatively by examining the consistency of findings across studies. Studies with larger sample sizes or more rigorous designs were given more weight in the interpretation of results. Potential biases such as publication bias or unreported negative results were also considered, but no specific sensitivity analysis was performed to assess the impact on the overall conclusions. | The process is not explicitly written in the original text. |
| Reporting biases | 21 | Due to the nature of the studies included in this review, there was no formal assessment of reporting bias (e.g., publication bias) through tools like funnel plots or statistical tests. However, efforts were made to minimize reporting bias by including a comprehensive search strategy and considering both published and unpublished studies when available. All relevant outcomes were reported if data were accessible. The possibility of missing or unpublished results, particularly those with null or negative findings, was acknowledged but not formally quantified. | Due to the nature of the data and available research, it was not explicitly explored. |
| Certainty of evidence | 22 | The certainty of the evidence for the outcomes assessed was generally considered low to moderate, reflecting the varied study designs and potential biases identified across the included studies. Given the cross-sectional nature of most studies, the lack of control for con-founders, and the potential for measurement bias, the confidence in the estimates of health impacts among esports athletes is limited. No formal grading of evidence (e.g., GRADE) was conducted, but the heterogeneity in study designs and methods suggests that further high-quality randomized controlled trials and longitudinal studies are needed to strengthen the evidence base and improve certainty in the outcomes. | The process is not explicitly written in the original text |
| **DISCUSSION** | | |  |
| Discussion | 23a | The findings from this review suggest that esports athletes, particularly professional and elite players, face a range of health issues, including musculoskeletal discomfort, eye strain, mental health challenges, and sleep disturbances. These results align with previous studies that have highlighted similar concerns among athletes in other types of competitive sports and gaming environments (Kurniawan et al., 2024; Ekefjärd et al., 2024). Notably, many of the issues identified—such as fatigue, stress, and physical discomfort—are comparable to those observed in athletes in physically demanding sports, suggesting a need for health management strategies that are tailored to the unique demands of esports.  Despite some variation in study designs and methodologies, the overall pattern of health risks found in this review is consistent with the broader literature on professional esports. However, the lack of high-quality longitudinal studies and the cross-sectional nature of most of the included studies limit our ability to draw causal conclusions. Future research, particularly longitudinal studies with more rigorous controls, is needed to better understand the long-term health impacts of esports participation and to develop effective preventive measures. | Manuscript document, line 157-339. |
|  | 23b | The evidence included in this review has several limitations that should be considered when interpreting the results. First, many of the studies were cross-sectional in design, limiting the ability to draw causal inferences about the health impacts of esports participation. The majority of the studies also relied on self-reported data, which can introduce biases, such as underreporting or overreporting of symptoms and behaviors, especially in studies that focused on physical or mental health outcomes.  Another limitation is the variability in the methodologies used across the included studies. While some studies used structured questionnaires and surveys, others relied on interviews or observational approaches, leading to differences in the way health outcomes were assessed. This heterogeneity in study designs may affect the comparability of results and contribute to some degree of measurement bias.  Additionally, there was a lack of long-term follow-up data, with most studies examining short-term health effects. This means that the long-term health risks associated with esports, such as chronic musculoskeletal problems or mental health issues, remain underexplored. The absence of studies with diverse populations also limits the generalizability of the findings to different esports disciplines or geographic regions.  Finally, the risk of publication bias cannot be ruled out, as studies with positive findings regarding health issues may be more likely to be published, while studies with null or negative results might remain unpublished. | The process is not explicitly written in the original text |
|  | 23c | Several limitations in the review process should be noted. First, there may have been selection bias in the included studies, as studies published in languages other than English or unpublished studies might have been missed. Additionally, the review only included studies up to a specific cutoff date, so more recent research was not considered.  Another limitation is the assessment of risk of bias. Some studies lacked detailed methodological information, such as randomization or blinding procedures, which may have affected the reliability of our bias assessments.  Due to the heterogeneity in study designs and outcome measures, we were unable to conduct a meta-analysis, limiting the ability to quantify the effects. The narrative synthesis may not fully capture the diversity of health outcomes or potential moderators in esports athletes.  Finally, inconsistencies in how health outcomes were reported and the exclusion of non-English studies may limit the generalizability of the findings. |  |
|  | 23d | **Practice:** Esports professionals should prioritize physical and mental health by incorporating health screenings, ergonomic practices, and mental wellness programs. Regular exercise and balanced lifestyles are essential for preventing injuries and improving overall well-being.  **Policy:** Esports organizations should develop health guidelines, including regular health checks, screen time limits, and support for mental health. Policy efforts should focus on ensuring the well-being of athletes through funding and health initiatives.  **Future Research:** Further studies should focus on standardized health assessments, long-term effects of esports, and the impact of specific interventions like ergonomic training. Research should also explore health disparities across different player groups and regions to develop targeted solutions. | The process is not explicitly written in the original text |
| **OTHER INFORMATION** | | |  |
| Registration and protocol | 24a | This systematic review was not registered. | The process is not explicitly written in the original text |
|  | 24b | A protocol for this review was not prepared. |  |
|  | 24c | No modification. |  |
| Support | 25 | No funding or other support for this review. |  |
| Competing interests | 26 | No competing interests |  |
| Availability of data, code and other materials | 27 | Data from included studies, data used in all analyses, and other materials used in the review are publicly available and can be found in paper databases such as Google Scholar |  |

*From:*  Page MJ, McKenzie JE, Bossuyt PM, Boutron I, Hoffmann TC, Mulrow CD, et al. The PRISMA 2020 statement: an updated guideline for reporting systematic reviews. BMJ 2021;372:n71. doi: 10.1136/bmj.n71. This work is licensed under CC BY 4.0. To view a copy of this license, visit <https://creativecommons.org/licenses/by/4.0/>
